# Parent-Reported Behavioural Changes in Children With Autism Spectrum Disorder During the COVID-19 Lockdown in Italy

**DOI:** 10.5334/cie.20

**Published:** 2020-09-16

**Authors:** Magda Di Renzo, Federico Bianchi Di Castelbianco, Elena Vanadia, Massimiliano Petrillo, Simona D’Errico, Lidia Racinaro, Monica Rea

**Affiliations:** 1 Institute of Ortofonologia, IT

**Keywords:** Autism Spectrum Disorder, COVID-19, quarantine, parents, clinical symptoms

## Abstract

Autism Spectrum Disorders (ASD) are characterized by impairments in social interaction and reciprocal communication. During a pandemic, when social distancing becomes mandatory for all, both parents and children are not supported in-person by their therapists and cannot participate in usual therapies. This study reports the main clinical changes experienced by parents of children with during the first month of lockdown during the COVID-19 pandemic in Italy. Using standardized questionnaires, the families of 63 ASD children were interviewed in February and April 2020. Findings showed an increase in restricted and repetitive behaviours in about 30% of the sample; also, motor restlessness and sleep disturbances increased, with greater irritability and mood dysregulation. On the other hand, no changes emerged in personal self-care autonomies, in taste/smell sensitivity, and in auto or other-directed aggression.

Despite the undeniable negative impact that lockdown can have on the psychosocial well-being of children, having maintained a continuity in supporting families, parental counselling (even if online and not in-person) helped families to redefine the meanings of behavioural changes of their children and to understand their adaptive functionality.

## Children With Autism During the Coronavirus Lockdown

The coronavirus COVID-19 pandemic in 2020 led to many governments mandating restrictive measures to be taken in order to prevent its wider spread. For parents and children, staying at home was one of those measures, including families with a child with Autism Spectrum Disorder (ASD). Before the pandemic in Italy, most children with ASD were included in more or less intensive rehabilitation programmes at home and in dedicated hospitals or institutions. During the pandemic, due to the social distancing, both families and ASD children were not supported in-person by their therapists and were not able to participate in external therapies (Narzisi, 2020). In fact, in Italy health facilities were told to provide only “emergency” medical care and, as a result, people with other health problems, including mental health disorders, have had to wait until the end of the emergency period to seek help, probably until October 2020. That is, because they are not considered to be in an “emergency” condition, patients with chronic mental health problems who need constant medical care have not been able to access proper care.

Cassidy et al. (2020), based on a roundtable discussion, published “An Expert Discussion on Autism in the COVID-19 Pandemic,” which, among other things, summarizes the main international ASD-related organizations that are working on creating guidelines in their respective countries (e.g., the National Autistic Society in the UK, The Autism Society of America in the USA). Everyone agrees that dealing with uncertainty, changes in routine, loss of long-standing coping mechanisms, increased social isolation, and lack of connectedness are important risk factors caused by physical distancing. Added to this are the possible increases or flare-ups in co-occurring mental health issues, such as anxiety. Experts also fear that “rationing of health care resources may discriminate against people with disabilities by confusing measures of frailty with disability-related functional challenges” (Cassidy et al., 2020, p. 116).

In Italy, regardless of the type of therapeutic treatment, the rehabilitation programmes for children with ASD are usually also focused on parental support in order to better manage typical symptoms of autism and stimulate the child’s social and communication skills. Home quarantine as well as the closure of day care facilities are likely to have a negative impact on clinical outcomes, with a risk of aggravation of symptoms and even a relapse. Having to stay home not only slows progress in developing social skills, but also reduces self-reliance and self-confidence (Chaturvedi, 2020).

Further, some children with severe autistic symptoms may not understand what is going on, why their school is no longer accessible, why their routines are interrupted. Hence, it is possible that some of them will have difficulty adapting to changes in their environment. Thus, the intrinsic characteristics of autism (impairments in social communication and interaction, restricted and repetitive behaviours, interests, or activities) and the frequent co-existence of neurological, psychiatric, and medical co-morbidities (Christensen et al., 2018) make individuals with autism a more vulnerable population that needs maximum attention in the context of the prevention and control strategies of the COVID-19 epidemic (Istituto Superiore di Sanità [ISS], 2020). The National Institute of Health (ISS, 2020) issued important recommendations for describing the measures currently in place (e.g., social distancing) and even the most negative ongoing events (symptomatic parent at home or hospitalization) to children with ASD through the use of concrete terminology, avoidance of abstract sentences or metaphors, and/or use of augmentative and alternative communication interventions (Logan, Iacono, & Trembath, 2017). Some children may find it difficult to articulate how they feel about unexpected changes. For others, communication impairments may be associated with problems of receptive and expressive communication, limited verbal, or nonverbal skills, and perspective and/or social communication deficits.

Fear, frustration, and worry can be expressed through behaviours such as changes in sleep/wake rhythms or eating patterns, an increase in repetitive behaviours, excessive rumination, an increase in agitation or irritability, or a decrease in self-care (Hume et al., 2020). Further, many individuals with ASD use electronic devices (such as television, tablet, smartphone) more often and for longer periods of the day, making it difficult to stop using them or to switch from one activity to another. The rigidity and inflexibility of some individuals with autism can make these transitions particularly problematic (ISS, 2020).

The objective of this study was to investigate the main changes perceived by parents in their child’s autistic symptomatology during the first month of home quarantine imposed by Italian governmental decrees to deal with COVID-19. We specifically wanted to (a) to verify the presence of improvements or worsening of the typical symptoms of autism (e.g. restricted and repetitive behaviours, mannerisms and stereotypes); (b) investigate changes in children’s self-care skills; and (c) verify changes in arousal (hyperactivity, fears induced by new situations, and problems with sleeping), in aggressiveness (aggressiveness and irritability), and the sensory profile (olfactory and gustatory sensitivity).

## Method

### Participants

The study included 63 families and their children with ASD; eight females and 55 males aged between 2.7 and 9.4 years old (see Table 1). Of the children, 36.5% had a mild level of diagnostic severity; 38.1% a moderate level, and 25.4% a severe level. With reference to stages of language development, 28.6% had no language, 22.2% had holophrastic speech, 20.6% had telegraphic speech, 28.6% had a complete phrasal structure. Further, 92% were of Italian origin; the families of the remaining 8% were from Afghanistan, Cameroon, Philippines, Peru, and Romania. A total of 33% of the children were only children, 57% had a brother/sister, 8% had two siblings, and 2% had four siblings.

**Table 1:** Characteristics of the Study Sample.

	*Mean* (*SD*)	Range

Child age, years	5.9 (1.7)	2.7–9.4
Child age when beginning rherapy, months	34 (7.8)	21–55
Treatment duration, months	38.9 (20.1)	4–82
Child intelligence quotient	84.3 (16.3)	44–112

All the children followed a therapeutic approach (DERBBI model) for ASD (Di Renzo et al., 2020). DERBBI – Developmental, Emotional Regulation and Body-Based Intervention – is focused on constructing communicative interaction with children; it is mediated by a therapist and the caregiver, who help the child to regulate his or her own reactions when confronted with external or internal stimuli that may be perceived as disturbing or harmful. The specific characteristic of the intervention is the use of the therapist’s body as a communication tool, in order to enrich an emotional exchange in the dyad (for further details, see Di Renzo et al., 2020). All children ceased therapy March 10, 2020, because of government regulations related to minimize the spread of COVID-19.

### Procedure

The parents of children with ASD were involved in this study. At the time of the study, the children had been participating in the Institute of Ortofonologia (Rome) rehabilitation projects for about three years after receiving a diagnosis of ASD in accredited private centres or public programs in Rome (Italy). From March to April 2020, the children had to cease attending the rehabilitation programme; instead, their parents conducted weekly telephone and Skype counselling meetings with psychologists.

For the study, the parents were contacted by phone, and a subsequent 40-minute telephone appointment was scheduled. Psychologists made the calls between April 10 and 14, 2020 (T1). They explained to the parents the aims of the research and that during the calls they would discuss the general well-being of their children through a series of questionnaires that had been presented to them in February 2020 (T0) during periodic follow-up meetings. No parent refused to participate in the research; all telephone interviews were conducted with mothers.

### Instruments

**ABAS-II.** The Adaptive Behaviour Assessment System-II provides an individualized measure of adaptive behaviour. The parent report of the ABAS-II (Harrison & Oakland, 2003) used in this study addresses nine adaptive skill areas (*M* = 10; *SD* = 3). In this study, the parents were given the Self-Care subscale in the form of a structured interview.

**ASDBI.** The ASD Behaviour Inventory (Cohen & Sudhalter, 2005) is a standardized rating scale for parents; it was designed to include separate subscales that independently address different types of maladaptive or adaptive behaviours. In this study, the following maladaptive subscales (higher scores indicative of more behaviour problems) were administered: (a) Arousal Problems, assessing hyperactivity, fears induced by new situations and problems with sleeping; and (b) Aggressiveness, assessing both self and other-directed aggression, along with general moodiness and irritability.

**SSP.** The Short Sensory Profile (McIntosh, Miller, & Shyu, 1999) assesses sensory symptoms in seven domains with 38 items to determine how the child modulates sensory inputs through the sensory systems and which behavioural and emotional responses are associated with sensory processing. In this study only the Taste/Smell Sensitivity subscale was administered. Parents were asked to indicate how often their child showed sensory behaviours. Scores are assigned on a five-point Likert scale ranging from “always” = 0 to “never” = 4. Low scores are indicative of frequent dysfunctional behaviours.

### Statistical Analyses

In order to evaluate variations in children’s scores between T0 (February 2020) and T1 (April 2020), analyses of unifactorial (ANOVA) and multivariate (MANOVA) variance for repeated measurements were conducted. Effect size was calculated using the partial eta squared, whereby η^2^ = 0.02 is considered a small effect, 0.13 a medium effect, and 0.23 a large effect (Pierce, Block, & Aguinis, 2004). In order to analyse changes over time of the measures based on categorical variables, an analysis of the chi-square was also carried out. The level of significance was set at *p* < 0.05. All statistical analyses were performed using the software version 21.0 of SPSS.

## Results

### Changes in Autistic Symptoms

The first goal of the study was to verify the presence of improvements or worsening of the typical symptoms of autism. Here 33.6% of parents (21 out of 63) reported intensification of autistic typical symptoms in the last month; 14% (9 out of 63) reported an increase in restricted and repetitive behaviours, 1.6% (1 out of 63) an increase in mannerisms, 14% (9 out of 63) an increase in motor stereotypies, and 3.2% (2 out of 63) an increase in vocal stereotypes.

### Changes in Self-Care Autonomies

The second aim was to verify changes in self-care skills (ABAS-II subscale). Parents did not report any changes in the autonomies related to the use of the toilet, in washing or dressing in the last month (*F* = 3.169; *p* = .08) (see Table 2).

**Table 2:** Difference Between the Averages of the Scores on the ABAS-II Self-Care Subscale, the ASDBI Subscales, and the SSP.

Test	Subscale	T0	T1	*F*	*P*	η^2^

ABAS-II	Self-Care	2.1	2.2	3.169	.08	/
ASDBI	Hyperactivity	1.0	1.4	49.00	<.01	.44
	Fear of New situations	0.7	0.9	11.698	<.01	.16
	Sleep Regulation Problems	0.4	0.8	15.645	<.01	.20
	Moodiness	0.4	0.6	11.737	<.01	.16
	Irritability	0.9	1.3	26.481	<.01	.30
	Self-Directed Aggression	1.6	1.7	0.219	.64	/
	Other-Directed Aggression	0.4	0.5	3.189	.08	/
SSP	Taste/Smell Sensitivity	3.6	3.5	3.253	.08	/

*Note*: ABAS-II: Adaptive Behavior Assessment System, Second Edition. ASDBI: Autism Spectrum Disorder Behavior Inventory; SSP: Short Sensory Profile. T0 = February 2020; T1 = April 2020.

### Maladaptive and Sensory Problems

The third aim was to verify changes in maladaptive problems. As shown in Table 2, the scores at the ASDBI subscales significantly increased, thus indicating a worsening in Arousal Problems, specifically in Hyperactive Behaviours, such as motor restlessness, agitation, moving back and forth in the room (*F* = 49.00; *p* < .01; η^2^ = .44), in Fears induced by new situations (*F* = 11.698; *p* < .01; η^2^ = .16), and in Sleep Regulation Problems, such as difficulty falling asleep, nocturnal awakenings, and difficulty in waking up (*F* = 15.645; *p* < .01; η^2^ = .20).

Data analysis also revealed significant worsening in Aggressiveness, specifically in Moodiness (the child gets scared for no apparent reason, gets angry or cries suddenly, changes his mood quickly) (*F* = 11.737; *p* < .01; η^2^ = .16), in Irritability (the child has a tantrum, is hard to please) (*F* = 26.481; *p* < .01; η^2^ = .30). In contrast, no significant changes were found in the Self- and Other-Directed Aggression subscales (hitting, scratching, biting themselves or others). Finally, parents did not perceive significant changes in their children in the Taste/Smell Sensitivity subscale of the Short Sensory Profile (see Table 2).

The variables “age of the child” (*p* = .82), “severity of autistic symptomatology” (*p* = .53), and “intelligence quotient” (*p* = .52) were included as covariates, but none of these was significant, indicating that the variations detected over time were independent from these variables.

### Qualitative Responses

From the analysis of the frequency distribution of individual items, it emerges that in the Hyperactive subscale, the percentage of children who “often” showed problems significantly increased on all four items included in the subscale (see Table 3). Even in the Fears induced by new situations and Sleep Regulation Problems, the percentage of children who “often” showed difficulties, both in falling asleep and awakening phase, increased significantly.

**Table 3:** Difference Between Response Rates to the ASDBI Subscales.

Subscale	Item	Answer	T0 (%)	T1 (%)	Chi square	P

Hyperactivity	Restless	N/A	61.9	38.1	20.71	.001
		S/t	36.5	25.4		
		Oft.	1.6	36.5		
	Fidgets	N/A	63.5	34.9	11.64	.01
		S/t	36.5	34.9		
		Oft.	0.0	30.2		
	Climbs on furniture	N/A	71.4	66.7	78.19	.001
		S/t	17.5	14.3		
		Oft.	11.1	19.0		
	Wanders around room	N/A	60.3	49.2	53.54	.001
		S/t	34.9	27.0		
		Oft.	4.8	23.8		
Fear of New Situations	Becomes upset when things don’t occur at their usual times	N/A	88.9	826	32.41	.001
		S/t	9.5	11.1		
		Oft.	1.6	6.3		
	Resists changing from one activity to another	N/A	57.2	46.1	60.39	.001
		S/t	36.5	34.9		
		Oft.	6.3	19		
	Becomes upset when own schedule or order of the routine is changed	N/A	79.3	68.3	61.50	.001
		S/t	15.9	20.6		
		Oft.	4.8	11.1		
	Resists changing own location in room	N/A	81	69.9	63.91	.001
		S/t	12.7	19		
		Oft.	6.3	11.1		
Sleep Regulation Problems	Difficulty falling asleep	N/A	88.9	68.2	19.70	.001
		S/t	9.5	14.3		
		Oft.	1.6	17.5		
	Awakens one or more times at night	N/A	81.0	68.2	50.77	.001
		S/t	12.7	15.9		
		Oft.	6.3	15.9		
	Awakens unusually early and stays awake the rest of the day	N/A	87.3	76.2	62.26	.001
		S/t	7.9	12.7		
		Oft.	4.8	11.1		
	Difficulty awakening at morning	N/A	96.8	87.3	11.28	.01
		S/t	3.2	7.9		
		Oft.	0	4.8		
Moodiness	Becomes fearful for no reason	N/A	90.5	84.1	38.68	.001
		S/t	9.5	11.1		
		Oft.	0	4.8		
	Cries for no reason	N/A	98.4	93.6	/	ns
		S/t	1.6	4.8		
		Oft.	0	1.6		
	Angry for no reason	N/A	87.3	85.8	/	ns
		S/t	7.9	6.3		
		Oft.	4.8	7.9		
	Shift in mood quick	N/A	76.2	66.7	56.10	.001
		S/t	19	19		
		Oft.	4.8	14.3		
Irritability	Cranky	N/A	63.5	38.1	34.21	.001
		S/t	30.2	36.5		
		Oft.	6.3	25.4		
	Difficult to please	N/A	79.3	69.8	53.00	.001
		S/t	15.9	15.9		
		Oft.	4.8	14.3		
	Takes a long time to calm down when upset	N/A	77.8	71.4	67.02	.001
		S/t	15.9	15.9		
		Oft.	6.3	12.7		
	Easily frustrated	N/A	65	50.8	32.61	.001
		S/t	30.2	28.6		
		Oft.	4.8	20.6		

*Note*: ASDBI: Autism Spectrum Disorder Behavior Inventory; T0: February 2020; T1: April 2020; N/A: Never/Almost Never; S/t: Sometimes; Oft.: Often.

Finally, in the Moodiness and Irritability areas, the percentage of children who “often” showed difficulty significantly increased, especially with respect to becoming fearful for no reason and significantly changing one’s mood.

In addition, qualitative data from interviews with parents into the possible psychosocial impact of the pandemic on their children highlighted that 19 parents (about 30% of the total sample) reported improvements in the communicative-relational domain; parents of five children (out of 42 who had more than one child) reported improvements in the quality of relationships and play between siblings.

Further progress observed by parents during this period included both verbal communication, such as the appearance of single new words or initial verbalizations, and nonverbal communications, such as greater responsiveness in comprehension or the emergence of communicative gestures, a more present visual engagement, and greater attention to and engagement in activities such as homework, drawing, or playing.

## Discussion and Conclusion

In the period, March-April 2020, during the coronavirus (COVID-19) pandemic, physical distancing measures were implemented in Italy, thereby disrupting routines and reducing access to services for families and children with ASD. One month after the beginning of the restrictive measures announced by the Italian government, our findings showed an increase of restricted, repetitive behaviours, mannerisms, and stereotypes in about 33% of the children in the sample. This finding is similar to that reported by Sprang and Silman (2013), who found that 30% of isolated or quarantined children showed signs of post-traumatic stress disorder during the 2009 H1-N1 pandemic in the United States. On the other hand, the increase in stereotyped behaviours could also be interpreted as a sign of the children’s need to anchor themselves to a known pattern of behaviour (though apparently not functional), which could have a soothing value when daily routines were abruptly interrupted and the children had to adjust to a new and sudden adaptation, both external (social isolation and full-time coexistence with parents) and internal (managing emotions, such as anger or sadness).

As Tribulato (2014) argued, “ritual repetition is an effective tool for reducing anxiety … Through stereotypies, children with ASD try to reduce and counteract the anxieties, fears and internal conflicts … In fact, when the child’s anxiety and suffering decrease, this type of symptoms is greatly attenuated …. For this reason, it is absolutely useless and counterproductive to struggle to limit or try to extinguish these signs of suffering, through reproaches or negative reinforcements. It is much better to commit to offering the child a more peaceful, joyful and dialoguing environment” (pp. 50–51).

In the present study, parents perceived a significant increase in their children’s difficulty in the areas of Hyperactivity and Fear toward new situations, expressed in terms of sensory-motor agitation and restlessness. This could be related to the absence of planned routines; in fact, according to parents’ reports, in the months preceding the lockdown, routine activities (school, therapy, extracurricular activities) had helped them contain and manage the conduct of their children.

At the same time, a worsening in the area of sleep regulation was found, showing a significant amount of nocturnal and/or early awakenings, agitation, and difficulty in falling asleep, associated in some cases with the need and/or desire of the children to go back to sleeping with their parents despite previously having developed the ability to sleeping alone in their own bed. Problems related to sleep regulation were also reported by other authors (Altena et al., 2020), who declared that potential for sleep problems to emerge or worsen during periods like this is high. This may be particularly true for children with neurodevelopmental conditions (including attention-deficit/hyperactivity disorder and ASD), who may be particularly vulnerable to disturbed sleep during this time of great change and uncertainty (Becker & Gregory, 2020). In addition, it should be emphasized that less or poor sleep may leads to greater attentional difficulties and increased restlessness, resulting in behaviours that mimic emotional dysregulation (Golberstein, Wen, & Miller, 2020).

In our study, participating parents perceived greater variability in the moodiness and irritability of their children, reporting an increase in apparently unmotivated fears and important difficulties in being comforted by the caregiver. Nevertheless, a very small percentage of children showed the emergence or intensification of self- and other-directed aggressive behaviours; this could be related to the increased tendency to express emotions or mood changes openly, which may represent a protective factor towards dysfunctional behaviours.

It is important to point out that the children’s self-care skill did not worsen, so that the skills acquired before the pandemic, supported by parents, were not lost. A further noteworthy finding is the absence of a correlation between symptomatic behaviours and intelligence quotient; that is, although the children in the present study were very heterogeneous with respect to their IQ scores, this did not seem to have affected any changes in symptoms during this first month of lockdown. This suggests that cognitive functions are not represented by a single performance, as expressed in a standardized test score, but may have an adaptive meaning; in this way, even those with a lower IQ score may be able to demonstrate coping strategies in a pandemic situation.

In light of the sudden change in habits that families and children with ASD experienced in the first month of lockdown, the preliminary results of this study underline the importance of parental support and parents’ need to have constant contact with professional support, even online if not possible in-person. During the counselling sessions, the psychologists found it important to redefine with parents the various meanings of “rehabilitation,” usually involving encouraging social interactions, reducing social distances, and promoting school inclusion.

The principal aim of therapeutic continuity in parental support was to accommodate caregivers’ previous and emerging vulnerabilities, but above all to help them make sense of the abnormal behaviours of their child. As one parent said, “Recently my son often got angry and cried with pain, especially when his dad went to work at the supermarket … only thanks to the longer time spent with him and to the fact that I was not scared at his strong reactions of anger, was I able to help my son say that he was crying because he was afraid that his dad would meet the virus.”

### Limitations

The study relied on parents’ perception of feelings and behaviours associated with a stressful experience, and, as such, responses are potentially flawed by recall bias and social desirability. Furthermore, the parents’ subjective level of distress may have interfered with their perceptive capacities regarding their child’s symptoms and functioning.

Also, the respondents who completed the online interview represent a self-selected group who may have had a special interest in the topic; therefore, generalizability of the results is limited.
